# Supplementary material for: AaSlt2 Is Required for Vegetative Growth, Stress Adaption, Infection Structure Formation, and Virulence in Alternaria alternata
Source: J Fungi (Basel). 2024 Nov 7;10(11):774. doi: 10.3390/jof10110774 (PMC11595810; doi:10.3390/jof10110774)
Supplement: Supplementary file 1 [file jof-10-00774-s001.zip › jof-3289570-supplementary.pdf]

Supplementary Material

Supplementary Figures

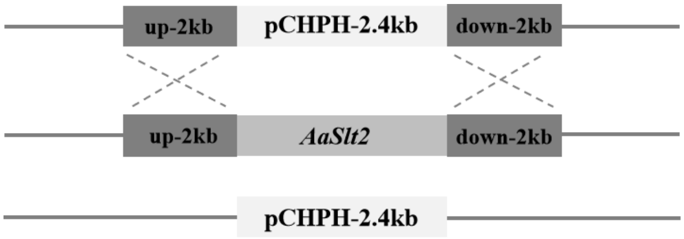

Supplementary Figure S1. Strategy of homologous recombination knockout of *AaSlt2*. The target fragment was recovered through a *hph* (Hygromycin B) resistance gene cassette.

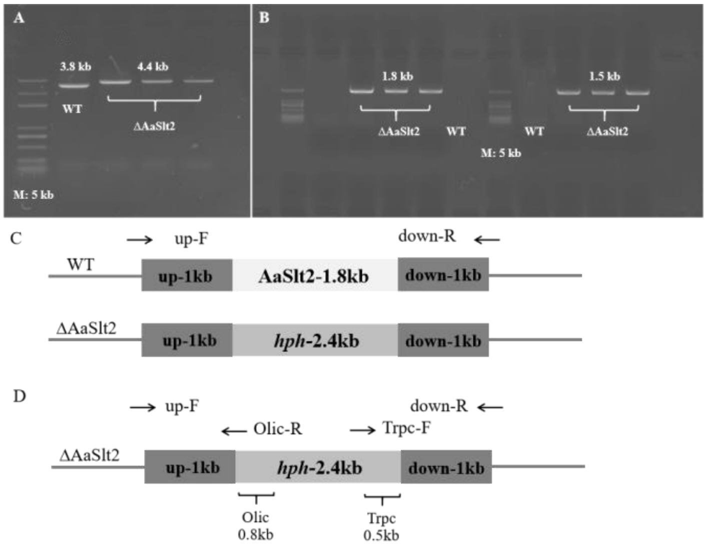

Supplementary Figure S2. Verification of positive transformant. (A) Validation of *AaSlt2* full-length. (B) Validationa of targete insertion. (C) Strategy of full-length verification of *AaSlt2* gene. (D) Strategy of targete insertion verification.

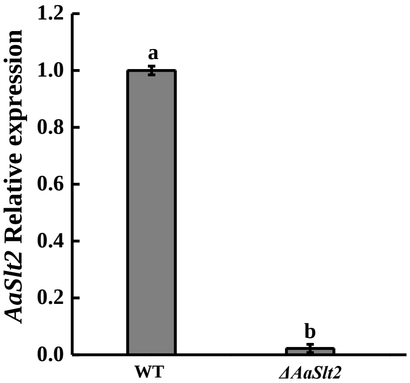

Supplementary Figure S3. *AaSlt2* gene expression analysis. Note: Bars indicate standard error (± SE). Different letters indicate significant differences ($p < 0.05$).

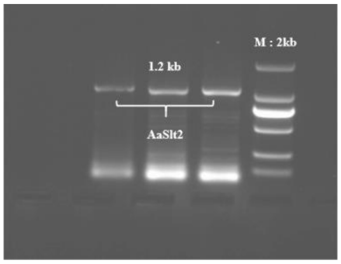

Supplementary Figure S4. Amplication of *AaSlt2* cDNA fragment for complementation.

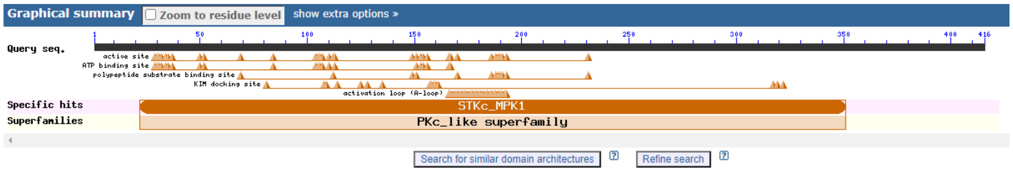

Supplementary Figure S5. Conserved domain of AaSlt2.

Supplementary Tables

Supplementary Table S1. Primers used for amplication of *AaSlt2*-up and *AaSlt2*-down.

Gene	Primer sequences (5'- 3')
AaSlt2-up	F: ACAGCTATGACCATGATTACGAATTCCAGAAGTGGCGATTGTGGGC
	R:GATCCCCGGGTACCGAGCTCGAATTCGCAGGAGTCGAGCAGCAGAG
AaSlt2-down	F:TTGCCTAACTCGGCGCGCCGAAGCTTAGAGCGTGAAGGATTTGGGTA
	R: GTAAAACGACGGCCAGTGCCAAGCTTATCACATCAGCAGCGGAGAA

Supplementary Table S2. Primers used for PCR amplification and qPCR in positive transformant.

Gene	Primer sequences (5'- 3')
OliC-R	CTGAAAGCACGAGATTCTTC
TrpC-F	TAGAGTAGATGCCGACCGG
AaSlt2-QF	TTGGTCTCGCAAGAGGTTTC
AaSlt2-QR	ACGTCGTGGCTTTCGTGTAG

Supplementary Table S3. Primers used for amplication of *AaSlt2* cDNA.

Gene	Primer sequences (5'- 3')
AaSlt2-NF	GCATGGACGAGCTGTACAAGGAGCTCATG GGCGACCTCGCCAACCG
AaSlt2-NR	ATGGAGCTATTAAATCACTATCTAGATCATCGCATG CGACCGTCAA

Supplementary Table S4. Primers used for PCR amplification in complementation transformants.

Gene	Primer sequences (5'- 3')
N-CX-1	CGACAACCACTACCTGAGCA
N-CX-2	TGAAGGGCGT ACTAGGGTTG

Supplementary Table S5. Primers used for qRT-PCR. *GAPDH* was used as the reference gene.

Gene	Primer sequences (5'- 3')
AaSlt2	F: TTGGTCTCGCAAGAGGTTTC
	R: ACGTCGTGGCTTTCGTGTAG
GAPDH	F: ATTGTCGCCGTAAACGACCC
	R: TTGACGGTCAGGTTGTTGCC
